# Relationship between dementia and depression: a case series

**DOI:** 10.1192/j.eurpsy.2023.1747

**Published:** 2023-07-19

**Authors:** A. Izquierdo De La Puente, P. del Sol Calderón, R. Fernández Fernádez, A. Rodríguez Rodriguez, M. Vizcaíno Da Silva, M. Martín García, O. Médez Gonzalez

**Affiliations:** 1 Psychiatry, Hospital Universitario Puerta de Hierro de Majadahonda, Majadahonda; 2 Psychiatry, Hospital Universitario Infanta Cristina, Parla; 3 Psychiatry, Hospital Universitario HM Mostoles, Mostoles; 4 Psychiatry, CSM Sal Carlos de El Escorial, El Escorial, Spain

## Abstract

**Introduction:**

Four cases are presented who debut with depressive episodes and after close follow-up, are diagnosed and treated for Alzheimer’s disease

**Objectives:**

The aim of this case series is to give a brief review of the depressive prodrome of dementia.

**Methods:**

Four women, aged 67-77 years, treated on an outpatient basis, consulted for depressive symptoms. In addition to affective symptoms such as apathy, lack of interest, sadness, increased emotional lability and anhedonia, all three reported cognitive impairment. In their follow-up after two years, they became progressively more dependent on their partners, with more memory lapses, forgetfulness and progressive loss of higher cognitive functions. With the progression of cognitive impairment, anxious symptoms have become increasingly present.

**Results:**

The mean age of the patients is 70 years. Two of them had an insidious onset of depressive symptoms, while the other two had a psychotic onset of depression. None of the patients had no previous history of depression. All four were started on antidepressant treatment with little response. Following the diagnosis of cognitive impairment, treatment was started with rivastigmine, with an adequate response.

**Conclusions:**

Dementia and depression are very common in the elderly. It appears that up to 40% of patients with dementia have depressive symptoms. It appears that depression in old age may actually be a prodromal symptom of dementia.

**Disclosure of Interest:**

None Declared

